# Unlocking the potential of *Polygonatum odoratum* protein hydrolysate: identification, characterization, and antidiabetic activity in HFD/STZ-induced type 2 diabetic mice

**DOI:** 10.3389/fnut.2026.1783967

**Published:** 2026-03-31

**Authors:** Yuying Ge, Yangqiannan Tang, Yuai Lu, Shiyi Hou, Zihao Dai, Ruiyao Xiong, Shuang Chen, Yamei Li, Bohou Xia

**Affiliations:** 1 School of Pharmacy, Hunan University of Chinese Medicine, Changsha, China; 2 Key Laboratory for Quality Evaluation of Bulk Herbs of Hunan Province, Hunan University of Chinese Medicine, Changsha, China

**Keywords:** HFD/STZ, Nrf2/Keap1, oxidative stress, *Polygonatum odoratum* protein hydrolysate, T2DM

## Abstract

*Polygonatum odoratum* is a traditional medicinal food used for diabetes management, yet most studies have focused on its polysaccharides and largely overlooked its protein fraction. Here, we investigated the antidiabetic activity and potential mechanisms of a *Polygonatum odoratum* protein hydrolysate (POP) for the first time. POP was produced by simulated gastrointestinal digestion and consisted mainly of oligopeptides (<1.5 kDa) enriched in leucine and valine. In HFD/STZ-induced type 2 diabetic mice, POP intervention significantly reduced hyperglycemia, improved glucose tolerance and insulin sensitivity, and alleviated dyslipidemia. Histological analyses showed that POP mitigated pancreatic *β*-cell injury, hepatic steatosis, and renal damage. POP also attenuated hepatic oxidative stress, as indicated by lower ROS and MDA levels and higher activities of antioxidant enzymes (SOD, CAT, and GSH-Px). Consistently, immunofluorescence indicated activation of the Nrf2/Keap1 pathway, with enhanced Nrf2 nuclear translocation and increased HO-1 expression. Therefore, POP improves glucose and lipid homeostasis and tissue damage in diabetic mice by enhancing Nrf2-dependent antioxidant defense mechanisms. These findings highlight the underexplored value of the P. odoratum protein fraction and support POP as a promising functional food ingredient for T2DM management.

## 1 Introduction

Type 2 diabetes mellitus (T2DM) is a major global health burden, affecting approximately 537 million adults worldwide (1, 2). It is characterized by chronic hyperglycemia driven by insulin resistance (IR) and progressive pancreatic *β*-cell dysfunction, and it is associated with a high risk of complications, including cardiovascular disease, nephropathy, and neuropathy (3). Current clinical management relies on lifestyle intervention and multiple pharmacological options, such as metformin, *α*-glucosidase inhibitors, GLP-1 receptor agonists, DPP-4 inhibitors, and sodium–glucose cotransporter 2 (SGLT2) inhibitors, which can effectively improve glycemic control and reduce cardiometabolic risk in many patients (4, 5). However, long-term adherence and accessibility remain challenges for some populations, and adverse effects (e.g., gastrointestinal intolerance, risk of hypoglycemia with certain agents, and other drug-specific side effects) may limit use or compliance (4, 5). Therefore, complementary strategies based on safe, diet-derived bioactives are of interest for long-term T2DM management.

Bioactive peptides from natural sources especially plant protein hydrolysates have attracted increasing attention as potential adjuncts for T2DM due to their favorable biocompatibility and multifunctional activities (6, 7). Emerging evidence suggests that such peptides may contribute to glucose homeostasis through several mechanisms, including inhibition of carbohydrate-digesting enzymes, modulation of insulin signaling, improvement of lipid metabolism, and attenuation of oxidative stress and inflammation (6, 7). As a medicinal food in Chinese tradition, *Polygonatum odoratum* (Mill.) Druce has historically been used to “quench thirst” and alleviate symptoms associated with diabetes (8). Previous studies have mainly focused on its polysaccharides, which show hypoglycemic activity (9, 10). Notably, the protein fraction of P. odoratum is also substantial, yet the antidiabetic potential of its protein-derived components particularly bioactive peptides remains underexplored. Addressing this gap may help to better utilize P. odoratum as a food resource for metabolic health.

Oxidative stress is closely involved in the development and progression of T2DM. Excessive reactive oxygen species (ROS) and impaired antioxidant defenses can aggravate IR and accelerate *β*-cell dysfunction (11, 12). The Keap1/Nrf2 pathway is a central regulator of cellular antioxidant responses; upon activation, Nrf2 translocates to the nucleus and induces cytoprotective genes, including heme oxygenase-1 (HO-1), thereby limiting oxidative damage (13, 14). Accordingly, natural compounds capable of modulating the Keap1/Nrf2 axis may provide a mechanistically meaningful approach for metabolic protection (15). However, whether a P. odoratum protein hydrolysate can alleviate T2DM related oxidative stress via this pathway has not been investigated.

Therefore, in this study, we first characterized the peptide profile of *Polygonatum odoratum* protein hydrolysate (POP). Then, using a high-fat diet (HFD) and streptozotocin (STZ)-induced T2DM mouse model, we comprehensively investigated the effects of POP on glycemic control, insulin sensitivity, lipid metabolism, and histopathological changes in key metabolic organs, including the pancreas, liver, and kidney. Furthermore, we explored the underlying mechanisms by assessing oxidative stress parameters and the expression of key proteins within the Nrf2/Keap1 pathway. This study provides the first evidence supporting POP as a novel functional food ingredient for T2DM intervention, effectively bridging the gap between traditional use and modern mechanistic understanding.

## 2 Materials and methods

### 2.1 Materials and reagents

*Polygonatum odoratum* (Mill.) Druce was procured from Hunan Gaoqiao Grand Market in Changsha, China, and was identified by Associate Professor Limin Gong of Hunan University of Chinese Medicine as the dried rhizome of Polygonatum odoratum (Mill.) Druce, belonging to the family Liliaceae. Petroleum ether (20220304) was obtained from Sinopharm Chemical Reagent Co., Ltd. Pepsin (porcine gastric mucosa, enzyme activity: 15500 u/g; RM19Y612) and trypsin [porcine pancreas, enzyme activity: 2512 U/mg (USP); RM21Y1216] were sourced from Shanghai Ryon Biological Technology Co., Ltd. Acetonitrile (F22MAB201) was acquired from Thermo Fisher Scientific. Streptozotocin (2230927005) was procured from Beijing Solarbio Science & Technology Co., Ltd. Recombinant Human Insulin (Powder; WH1722U151) was purchased from Wuhan Pricella Biotechnology Co., Ltd. HO-1 (ab68477), and Keap1 (ab119403) were obtained from Agtech (Shanghai) Trading Co., Ltd. Nrf2 (16396-1-AP) was acquired from Proteintech Group, Inc. Assay detection kits for glutathione peroxidase (GSH-Px; 20231012), catalase (CAT; 20240122), malondialdehyde (MDA; 20231102), and superoxide dismutase (SOD; 20231026) were procured from Nanjing Jiancheng Bioengineering Institute. The BCA assay detection kit was obtained from Saiwen Innovation (Beijing) Biotechnology Co., Ltd.

### 2.2 Preparation of POP

POP was prepared by simulated gastrointestinal digestion as previously described (16, 17). Degrease 500 g of *Polygonatum odoratum* (Mill.) Druce powder using petroleum ether (1:5, w/v), then dissolve the defatted powder in ultrapure water at a 1:10 (w/v) ratio. Adjust the pH to 10 with NaOH. Stir and filter, and the supernatant was collected. Adjust the pH to 4 with HCl and allow to stand at 4 °C for 12 h. Subsequently, centrifuge at 10,000 r/min for 10 min at 4 °C. Resuspend the precipitate, adjust to neutral pH, and obtain the crude extract via dialysis and freeze-drying (16). Resuspend this extract in ultrapure water at a 1:10 (w/v) ratio, add 5% pepsin, adjust pH to 2, and react at 37 °C for 2 h. The pH was then adjusted to 7.5, add 5% trypsin, and continue reacting at 37 °C for 4 h. The reaction was terminated by heating the solution in a boiling water bath (100 °C) for 15 min to remove trypsin and pepsin, then cooled to room temperature. The enzymatic digest was centrifuged at 10,000 r/min for 10 min at 4 °C. The supernatant was collected, freeze-dried to yield POP, with a POP yield of 10.97% (17).

### 2.3 *De novo* analysis of POP based on LC–MS/MS

The amino acid composition was determined using LC–MS/MS mass spectrometry, following reduction, alkylation, and desalting processes. Specifically, 1 mg of POP was solubilized in 0.1 mL of 50 mM NH_4_HCO_3_. An Easy-nLC 1,200/QExactive liquid chromatography-mass spectrometer (Thermo Fisher Scientific) was employed under the following liquid chromatography conditions: a Reprosil-Pur 120 C18-AQ 3 μm (100 μm i.d. × 180 mm) column, with mobile phase A consisting of 0.1% formic acid and mobile phase B comprising 0.1% formic acid and 80% acetonitrile, injected at a flow rate of 600 nL/min. The elution gradient was structured as follows: 0–3 min, 4–8% B; 3–89 min, 8–28% B; 89–109 min, 28–40% B; 109–110 min, 40–95% B; and 110–120 min, 95% B. The mass spectrometer was set to a full scanning range of m/z 100–1,500, with the primary mass spectrometer resolution adjusted to 70,000, AGC set to 3 × 10^6^, and maximum IT at 100 ms. The resolution of the secondary mass spectrometer was configured to 17,500, AGC at 1 × 10⁵, and maximum IT at 50 ms, while the peptide fragmentation collision energy was set to 28.

The raw mass spectrometry files were analyzed for peptide sequence resolution utilizing the PEAKS *De Novo* method employing the following search parameters: Fixed modifications included carbamidomethyl (C), while variable modifications comprised oxidation (M) and acetylation at the peptide N-terminus. A nonspecific enzyme was utilized, with a peptide mass tolerance set at 20 ppm and a fragment mass tolerance of 0.02 Da. A *De Novo* score of 80 or higher was established as the threshold for high-confidence screening, ensuring the reliability of peptide sequence inference.

### 2.4 Animal experiment design

**Figure 1:**
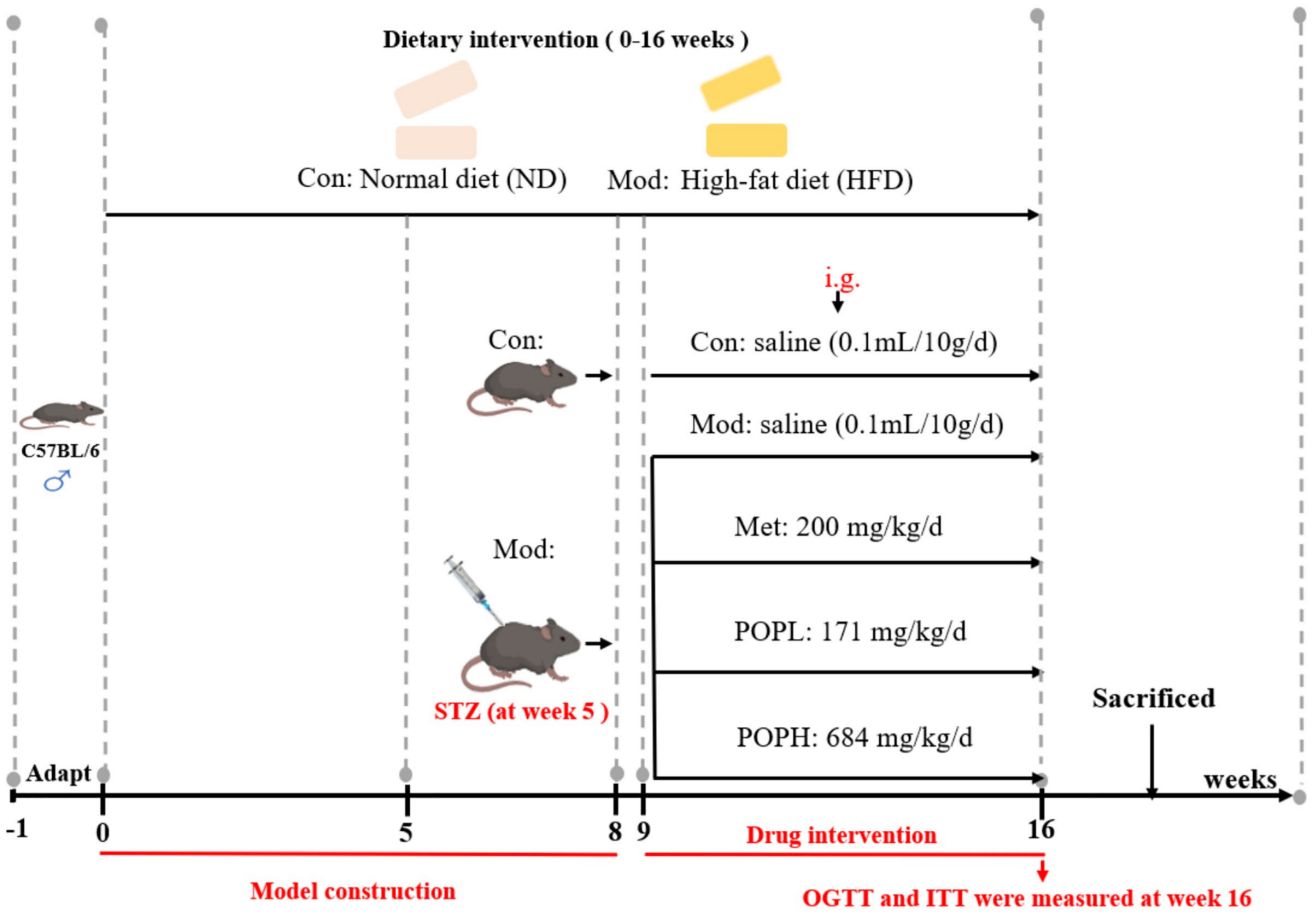
Flowchart of POP administration in T2DM mouse model.

The experimental program is depicted in Figure 1. This study complies with all relevant ethical standards, having received approval from the Ethics Committee of Hunan University of Chinese Medicine (LLBH-202307180001). A total of 40 male C57BL/6 J mice, aged 4 weeks and weighing between 16–18 g, were sourced from Hunan SJA Laboratory Animal Co., Ltd. and acclimatized at the Experimental Animal Center of Hunan University of Chinese Medicine. The mice were maintained under controlled environmental conditions: an ambient temperature of 22–24 °C, relative humidity of 60–65%, and a 12 h photoperiod, with unrestricted access to water and food. Following a 1-week acclimatization period, mice were randomly assigned to five groups using the random number table method (*n* = 8): Control group (Con), Model group (Mod), Metformin group (Met), POP Low dose group (POPL), and POP High dose group (POPH). The Con group was fed standard rodent chow (Caloric composition: 10% fat, 20% protein, 70% carbohydrate), while the Mod, Met, POPL and POPH groups were fed a 60% HFD (Caloric composition: 60% fat, 20% protein, 20% carbohydrate). Mice in the experimental group received intraperitoneal injections of STZ for 4 consecutive days during week 5, at a dose of 45 mg/kg/day (dissolved in citrate buffer at pH 4.5). Normal control group mice received an equivalent volume of physiological saline. The mice were fasted overnight before receiving STZ injection. Seventy-two hours after the final STZ injection, the mice’s RBG and FBG (fasting for 12 h) levels were measured. Mice meeting the screening criteria of three consecutive FBG readings exceeding 11.1 mmol/L and RBG levels above 16.7 mmol/L were deemed to have successfully established a diabetic model (18, 19). The Met, POPL, and POPH groups received either metformin (200 mg/kg/day dissolved in saline) or POP (171 or 684 mg/kg/day dissolved in saline), while the Con and Mod groups were given saline. Drug intervention commenced at the start of Week 9 of the experiment, with saline and medication administered via oral gavage. Following the final gavage administration, mice underwent a 12 h fasting period. Following isoflurane anesthesia (During anesthesia, the oxygen flow rate was set at 0.8–1.0 L/min, with the isoflurane vaporiser concentration rapidly adjusted to 3.5–4.0%. The induction process was completed within 2 min. Subsequently, the isoflurane concentration was adjusted to 1.5–2.0% to maintain the depth of surgical anesthesia.), blood samples were collected via the orbits (3,000 r/min for 20 min at 4 °C), and the supernatant was retained as serum. Kidney, liver, and pancreas tissues were harvested and weighed to determine the liver index, calculated using the formula: organ index = organ weight (mg)/body weight (g). Portions of the liver, kidney, and pancreas were fixed in 4% paraformaldehyde solution for subsequent histopathological examination and immunofluorescence analysis. The remaining tissues and serum were stored at −80 °C.

### 2.5 Fasting and random blood glucose testing

After successfully modeling, a standard glucose meter (GA-3, Sinocare Inc.) was employed to collect blood samples from the tails of the mice at a fixed time each week for the assessment of random blood glucose (RBG) and fasting blood glucose (FBG). The mice underwent a fasting period of 12 h prior to FBG testing.

### 2.6 Homeostasis model assessment-insulin resistance

The Homeostasis Model Assessment of Insulin Resistance (HOMA-IR) is one of the most widely utilized methods for evaluating insulin resistance (20). Serum insulin (INS) levels in mice were quantified using the INS ELISA research kit from WUHAN HUAMEI BIOTECH CO., LTD. The formulas for calculating HOMA-IR and the Homeostasis Model Assessment of *β*-cell function (HOMA-β) are as follows: HOMA-IR = FBG (mmol/L) × INS (nU/mL) / 22.5, and HOMA-β = 20 × INS (nU/mL) / [FBG (mmol/L)–3.5].

### 2.7 Oral glucose tolerance test and insulin tolerance test

In the sixteenth week, an oral glucose tolerance test (OGTT) was conducted to evaluate changes in glucose tolerance among the various groups of mice (21). Following a 12 h fasting period, blood samples were collected from the tail veins of the mice, and a standard glucose meter was utilized to measure blood glucose levels at baseline (0 min). Each group of mice received glucose via oral gavage at a dosage of 2 g/kg, with blood glucose measurements taken at 30, 60, 90, and 120 min post-administration. The recorded blood glucose values were plotted on the OGTT curve, and the area under the curve (AUC) was calculated using the trapezoidal rule. Additionally, an insulin tolerance test (ITT) was performed to assess changes in insulin tolerance among the groups (22). Following the same 12 h fasting period, blood was again collected from the tail veins, and baseline blood glucose was measured using a standard glucometer. Subsequently, the mice received an intraperitoneal injection of human recombinant insulin at a dose of 1 IU/kg, with blood glucose levels measured at 30, 60, 90, and 120 min thereafter. The blood glucose values were recorded, the ITT curves were plotted, and the AUC for the ITT curves was also calculated using the trapezoidal rule.

### 2.8 Biochemical assays

Serum total cholesterol (TC), triglycerides (TG), low-density lipoprotein cholesterol (LDL-C), high-density lipoprotein cholesterol (HDL-C), alanine aminotransferase (ALT), aspartate aminotransferase (AST), alkaline phosphatase (ALP), blood urea nitrogen (BUN), creatinine (Cr), and uric acid (UA) were measured using a fully automated hematology biochemistry analyzer (BK-280, Boke Biological Industry Co., Ltd.). Hemoglobin A1c (HbA1c) was determined using an HbA1c ELISA kit from Wuhan Huamei Biotech Co., Ltd. Liver tissues were collected and weighed, and pre-cooled PBS was added to a high-speed low-temperature tissue grinder (Retsch/MM 400, Wuhan Servicebio Technology Co., Ltd.) at a ratio of 1:9. The resulting liver tissue homogenate was centrifuged at 4 °C, 5000 r/min for 10 min, and the supernatant was collected. GSH-Px, CAT, MDA, and SOD were measured in the liver supernatants.

### 2.9 Reactive oxygen species detection

The livers fixed in a 4% paraformaldehyde solution were dehydrated, immersed in wax, embedded, sectioned using a Cryotome E (Thermo), rewarmed, and stained with DHE (PBS: DHE = 200:1 to 300:1). The samples were protected from light and incubated for 30 min at 37 °C. The nuclei were rinsed with PBS three times for 5 min each, followed by staining with DAPI for 10 min while protected from light. The slices were rinsed with PBS three times for 5 min each and sealed with an anti-fluorescence quenching agent. Finally, the sections were examined under a Nikon inverted fluorescence microscope (Nikon Eclipse Ti2, Nikon Corporation) for observation and image acquisition. The quantitative data regarding reactive ROS were analyzed using ImageJ software.

### 2.10 Pathologic testing of the liver, kidneys, and pancreas

Liver, kidney, and pancreas tissues were fixed in a 4% paraformaldehyde solution, followed by dehydration, wax embedding, and sectioning. The sections were stained with hematoxylin and eosin (H&E) and periodic acid-Schiff (PAS) using the protocols provided by the HE Staining Solution Kit (S191003) and PAS Staining Solution Kit (S191008) from Wuhan Pinuofei Biological Technology Co., Ltd. The stained slices were then examined under a Nikon inverted fluorescence microscope to observe the pathological changes in the pancreatic, hepatic, and renal tissues of the mice.

### 2.11 Immunofluorescence assays

Liver and pancreas tissue sections were deparaffinized and subjected to antigen retrieval. Subsequently, the sections were serum-sealed and incubated at 37 °C for 30 min. Primary antibodies were applied and incubated at 4 °C overnight. Following this, secondary antibodies were prepared and incubated at 37 °C for 1 h. Nuclei were stained with DAPI, protected from light, for 5 min, and the sections were washed three times with phosphate-buffered saline for 5 min each time. Finally, the sections were sealed with an anti-fluorescence sealer. Observations and image acquisition were conducted using a Nikon inverted fluorescence microscope. The quantitative histological staining data were analyzed using ImageJ software.

### 2.12 Statistical analysis

Data were processed using R statistical software, and results are presented as mean ± standard deviation (X̅ ± SD). Comparisons between groups were conducted using one-way ANOVA. The LSD method was employed for cases with homogeneous variance, while Dunnett’s T3 method was utilized for non-homogeneous variance. A repeated-measures design was applied for one-way ANOVA comparisons when the sphericity test results were satisfactory; otherwise, one-way comparisons were executed using the Greenhouse–Geisser correction. A significance level of *p* < 0.05 was adopted.

## 3 Results

### 3.1 LC–MS/MS based POP sequencing analysis

**Figure 2:**
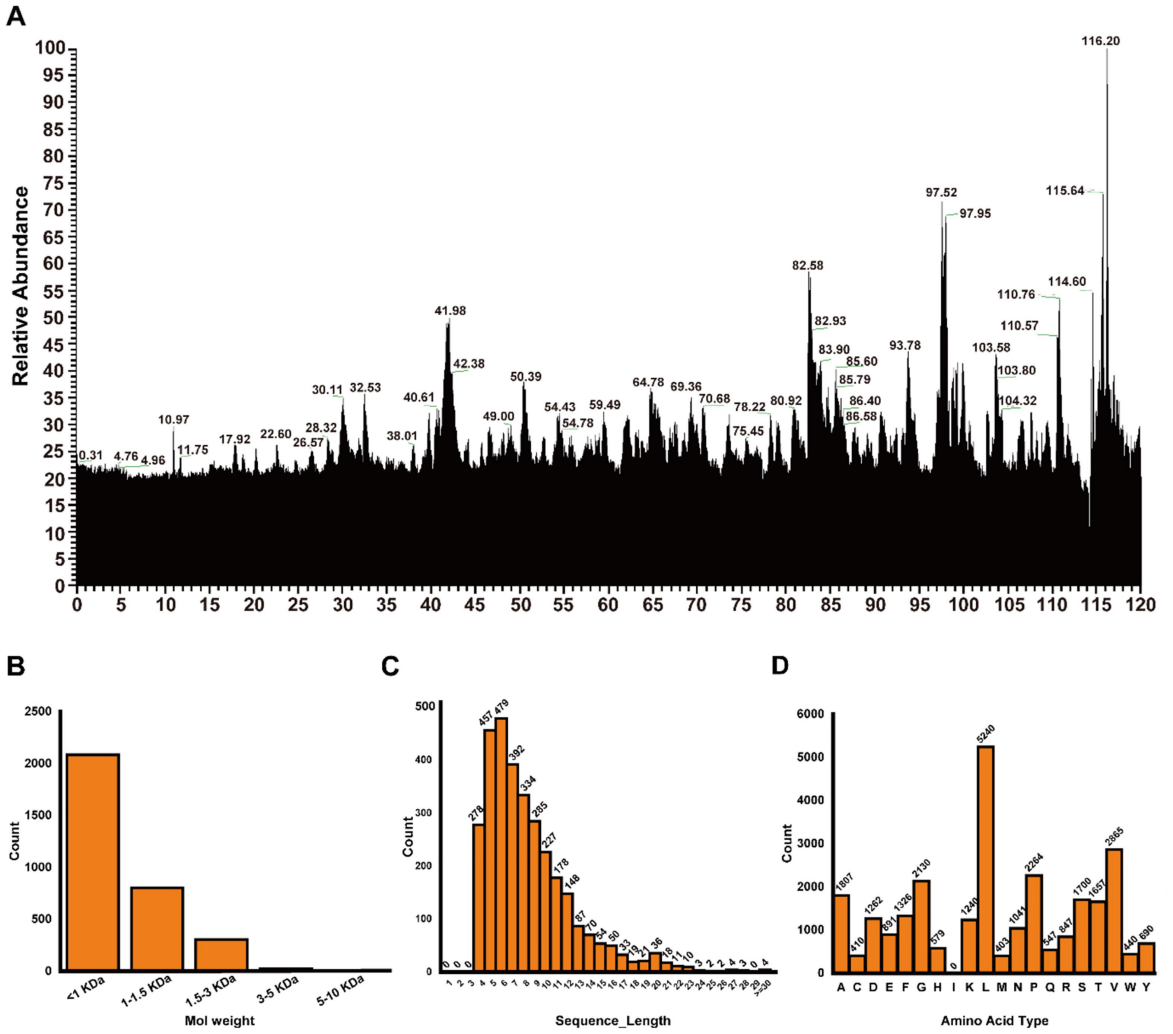
*De novo* sequencing analysis of POP through LC–MS/MS. **(A)** POP total ion current. **(B)** Distribution of different molecular weight peptide intervals in POP. **(C)** Peptide length distribution in POP. **(D)** Amino acid composition of peptides in POP.

Using LC–MS/MS technology, we performed *de novo* analysis of POP using PEAKS software (complete peptide spectrum analysis is provided in Supplementary file 1). The results indicate (as shown in Figure 2A) that peptides with molecular weights below 1.5 kDa constitute nearly 90% of the total peptide population, predominantly consisting of molecules smaller than 1 kDa (Figure 2B). Analysis of amino acid composition revealed that POP primarily consists of oligopeptides containing 4–9 amino acids (Figure 2C). Among these, the essential amino acids leucine (L) and valine (V), critical for human health, exhibited the highest proportions (Figure 2D).

### 3.2 Effects of POP on morphology, body weight, water intake and diet in STZ/HFD-induced T2DM mice

**Figure 3:**
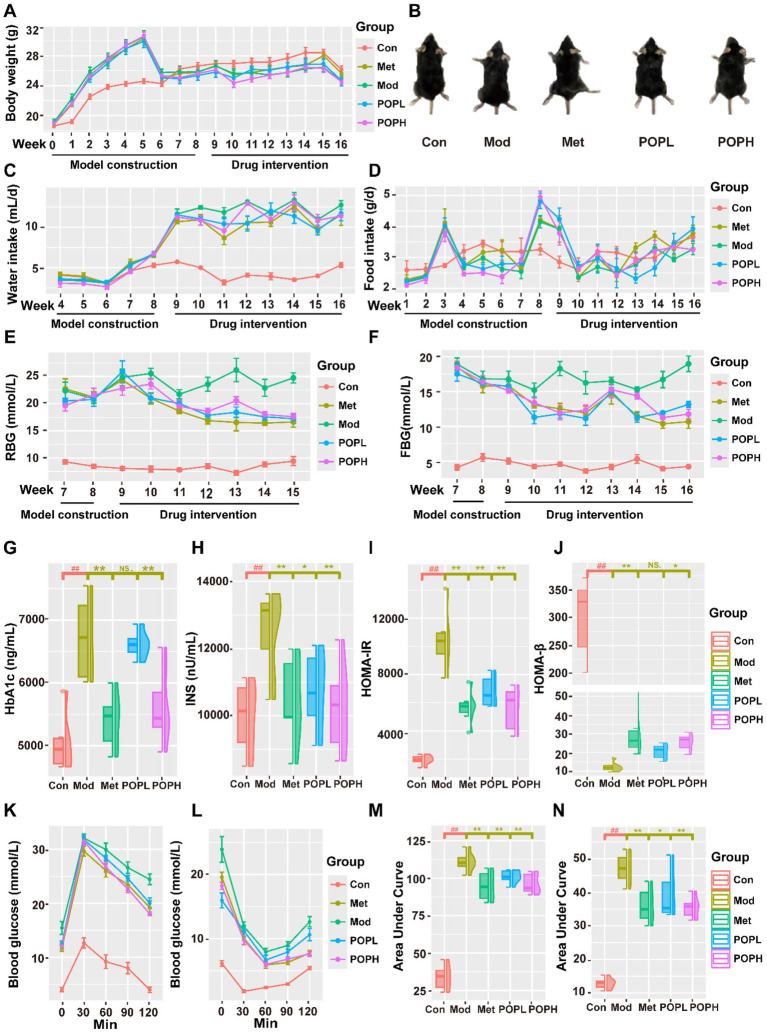
Effects of POP on morphology, body weight, water intake, dietary intake, blood glucose level and IR in T2DM mice. **(A)** Body weight. **(B)** Morphology of mice in each group. **(C)** Water intake. **(D)** Food intake. **(E)** RBG. **(F)** FBG. **(G)** HbA1C. **(H)** INS. **(I)** HOMA-IR. **(J)** HOMA-*β*. **(K)** Curve of OGTT at the end of the trial. **(L)** Curve of ITT at the end of the trial. **(M)** Areas under the curve of OGTT at end of the trial. **(N)** Areas under the curve of ITT at the end of the trial. Data are expressed as mean ± SD. #*p* < 0.05, ##*p* < 0.01, compared with the Con group; *p < 0.05, **p < 0.01, compared with the Mod group.

The general HFD model alone may only exhibit obesity and IR, necessitating the combination with STZ injections to fully replicate T2DM characterized by hyperglycemia and *β*-cell damage. Following the establishment of the STZ/HFD-induced T2DM mouse model, significant alterations in morphology, body weight, water intake, and dietary habits were observed. As illustrated in Figure 3A, T2DM mice exhibited a marked increase in body weight after 5 weeks of HFD feeding compared to the Con group. Following STZ injection, these mice exhibited symptoms including weight loss, lethargy, polyuria, increased food and water intake, and reduced activity levels. After the administration of POP, both POPH and POPL groups demonstrated a reduction in weight loss compared to the Mod group (Figure 3B). Furthermore, water intake significantly decreased in both the POPH and POPL groups relative to the Mod group (Figure 3C), while the effect on dietary intake was not significant (Figure 3D). In summary, POP appears to alleviate certain symptoms associated with T2DM, including morphological changes, polydipsia, and weight loss.

### 3.3 Effect of POP on blood glucose levels and IR in T2DM mice

The effects of various treatment groups on the blood glucose levels of mice during different intervention periods are illustrated in Figures 3E,F. Following the STZ injection, the RBG and FBG levels in each group of mice were significantly elevated compared to the Con group. Continuous testing revealed that RBG levels exceeded 16.7 mmol/L and FBG levels surpassed 11.1 mmol/L on three occasions, confirming the successful modeling of T2DM in mice. Drug administration commenced at the start of Week 9 of the experiment. Analysis of differences in FBG and RBG between experimental groups at Week 9 revealed no significant differences in baseline blood glucose levels among groups at the start of drug administration, with *p* values of 0.6094 for FBG and 0.6488 for RBG. During the drug treatment phase, both RBG and FBG levels in the Mod group remained stable but exhibited a slight increase. In contrast, both the POPL and POPH groups showed a significant reduction in RBG and FBG levels, with the decrease in RBG demonstrating a favorable time-dependence and approaching that of Met group. This suggests that POP may possess a superior hypoglycemic effect. The HbA1C level in the Mod group was significantly higher than that in the Con group (*p* < 0.01; Figure 3G). Conversely, the HbA1C level in the POPH-treated group was significantly lower than that in the Mod group (*p* < 0.01), indicating that POP has a sustained effect on alleviating hyperglycemia.

This study further evaluates the effects of POP on INS, HOMA-IR, HOMA-*β*, OGTT, and ITT. The results indicated that both INS and HOMA-IR were significantly elevated (*p* < 0.01; Figures 3H,I), while HOMA-*β* was significantly reduced (*p* < 0.01; Figure 3J) in the Mod group of mice compared to the Con group, suggesting that mice in the Mod group exhibited typical symptoms of IR. After POPL and POPH treatments, both INS and HOMA-IR levels in T2DM mice were significantly decreased (*p* < 0.01), accompanied by a notable increase in HOMA-β, indicating that POP significantly enhances insulin sensitivity and alleviates IR. Additionally, the OGTT and ITT results demonstrated significantly higher OGTT-AUC and ITT-AUC values in the Mod group, indicative of insulin resistance and impaired pancreatic β-cell function. However, POP treatment resulted in a dose-dependent downregulation of OGTT-AUC and ITT-AUC values (*p* < 0.01; Figures 3K–N). In conclusion, POP exhibits an antidiabetic effect, characterized by its ability to regulate blood glucose levels, improve glucose tolerance, and enhance insulin sensitivity in T2DM mice.

### 3.4 Protective effect of POP on islet function in T2DM mice

**Figure 4:**
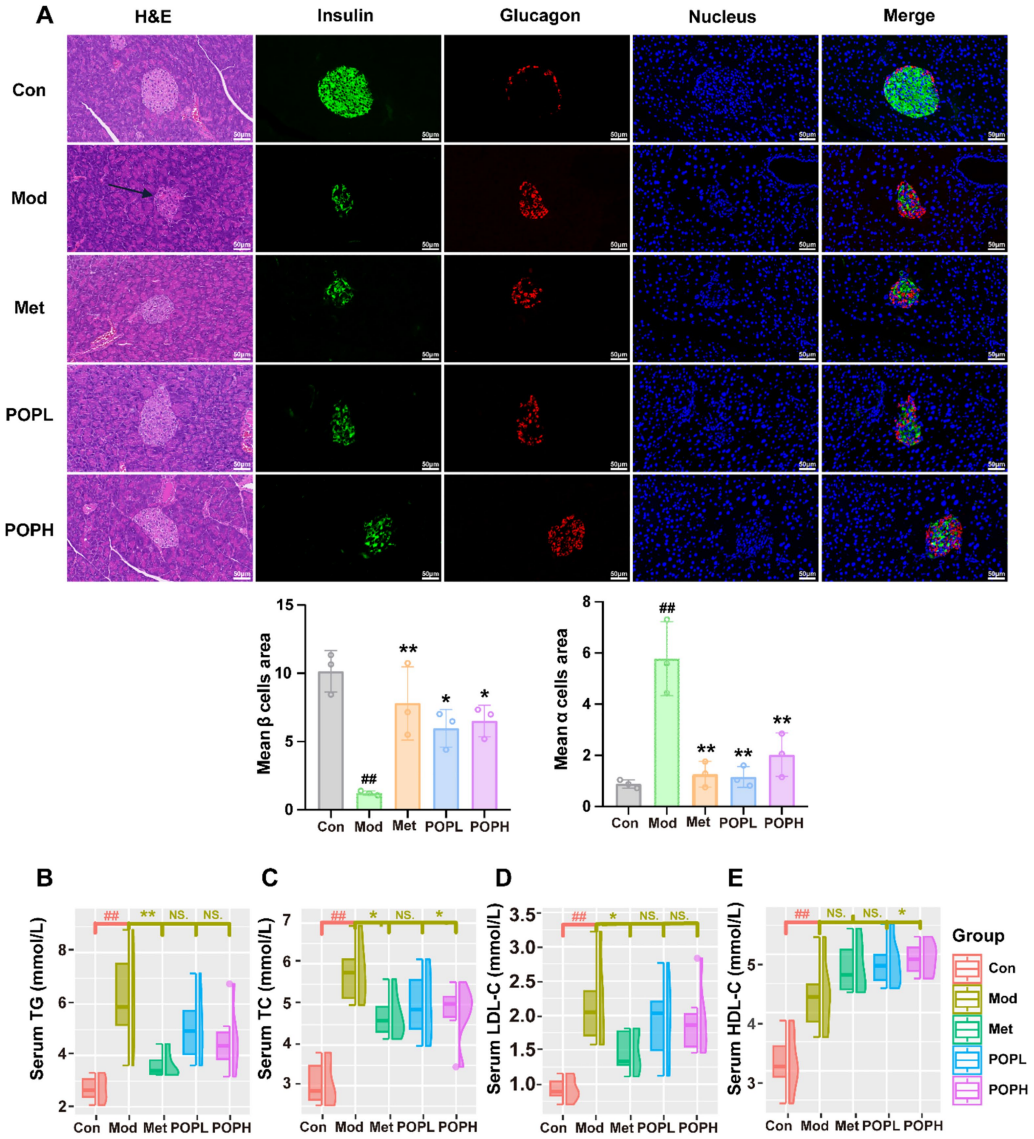
Protective effect of POP on pancreatic islet function and lipid metabolism in T2DM mice. **(A)** Immunofluorescence staining for Insulin (green), Glucagon (red), Nucleus (blue), and H&E staining of pancreatic islets. **(B)** Serum TG. **(C)** Serum TC. **(D)** Serum LDL-C. **(E)** Serum HDL-C. Data are expressed as mean ± SD. #*p* < 0.05, ##*p* < 0.01, compared with the Con group; *p < 0.05, **p < 0.01, compared with the Mod group.

The results of H&E staining of pancreatic tissues from mice across all groups in this study illustrated (Figure 4A) that the islets in the Mod group exhibited severe morphological alterations due to the induction of HFD and STZ, appearing atrophied, irregular in shape, and with indistinct borders compared to those in the Con group. Furthermore, results from the immunofluorescence assay indicated (Figure 4A) that the number of *β*-cells was significantly reduced while the number of *α*-cells was significantly increased in the islets of mice in the Mod group compared to the Con group. Notably, the deformation of islets and damage to pancreatic β-cells were significantly mitigated by treatment with POP and Met, resulting in a significant increase in the number of pancreatic β-cells (*p* < 0.05) and a significant decrease in the number of pancreatic α-cells (*p* < 0.01). Collectively, these findings suggest that POP significantly enhances islet morphology and β-cell function in T2DM mice, demonstrating a beneficial protective effect on the islets.

### 3.5 Effect of POP on lipid metabolism in T2DM mice

In this study, the results indicated that serum levels of TG, TC, LDL-C, and HDL-C were significantly elevated in the Mod group of mice compared to the Con group (*p* < 0.01; Figures 4B–E). Following POPH treatment, TC and HDL-C levels were significantly reduced (*p* < 0.05), while TC (*p* < 0.05), TG (*p* < 0.01), and LDL-C (*p* < 0.05) levels were significantly decreased in the Met group. No significant differences were observed for other indices, suggesting that POP has a notable effect on the regulation of lipid metabolism in T2DM mice.

### 3.6 Protective effect of POP on liver function in T2DM mice

**Figure 5:**
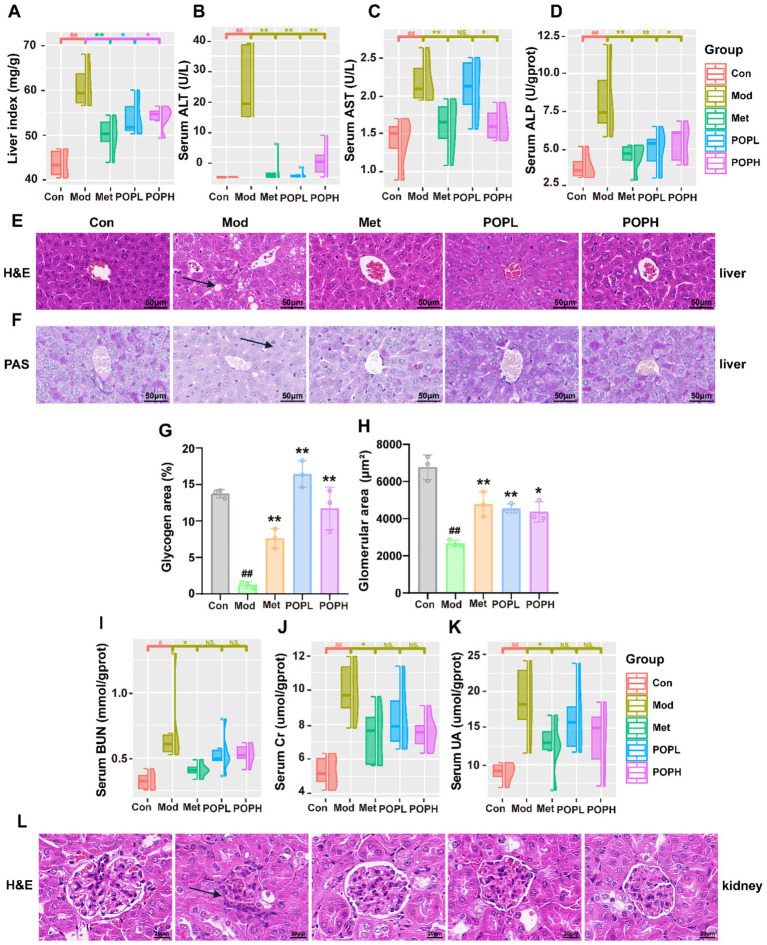
Protective effects of POP on liver and kidney function in T2DM mice. **(A)** Liver index. **(B)** Serum ALT. **(C)** Serum AST. **(D)** Serum ALP. **(E)** H&E staining to observe the pathological changes of the liver in each group of mice. **(F)** PAS staining to observe the pathological changes of the liver in each group of mice. **(G)** Glycogen area (%). **(H)** Glomerular area(μm^2^). **(I)** Serum BUN. **(J)** Serum Cr. **(K)** Serum UA. **(L)** H&E staining to observe the pathological changes of the kidney in each group of mice. Data are expressed as mean ± SD. #*p* < 0.05, ##*p* < 0.01, compared with the Con group; *p < 0.05, **p < 0.01, compared with the Mod group.

In this study, the organ index of the liver and the levels of ALT, AST, and ALP in the Mod group mice were significantly higher than those in the Con group (*p* < 0.01; Figures 5A–D), indicating hepatic dysfunction in high-fat diet-fed type 2 diabetic mice. Following treatment with POP and Met, the levels of ALT, AST, ALP, and the liver organ index were significantly reduced (*p* < 0.05). Liver H&E staining and PAS staining results (Figures 5E,F) further corroborated these findings. Compared to the control group, Mod group mice exhibited hepatocyte damage, abnormal hepatic lipid accumulation, and a markedly reduced capacity for glycogen synthesis and storage. Statistical analysis revealed that, compared with the Con group, Mod mice exhibited a significantly reduced proportion of glycogen area (*p* < 0.01). POP treatment significantly ameliorated these pathological alterations (*p* < 0.01; Figure 5G). In summary, this study demonstrates that POP effectively ameliorates hepatic dysfunction in T2DM mice, mitigates hepatic lipid accumulation, and enhances glycogen synthesis and storage capacity.

### 3.7 Protective effects of POP on renal function in T2DM mice

The results shown in Figures 5I–K indicate that, compared with the control group, BUN, CR and UA levels were significantly elevated in the Mod group mice (*p* < 0.05), suggesting renal impairment in T2DM mice. Following POP treatment, although BUN, CR and UA levels decreased, the reduction did not reach statistical significance. However, in the Met group, all three parameters were significantly reduced (*p* < 0.05). Furthermore, renal histological examination via H&E staining (Figure 5L) revealed abnormal glomerular morphology in the Mod group compared to the Con group, characterized by blurred glomerular borders, a marked reduction in normal tubules, in-creased atrophic tubules, thickened vascular walls, and interstitial inflammation. Statistical analysis revealed a significant reduction in glomerular area in the Mod group (*p* < 0.01). Treatment with Met, POPL, or POPH resulted in improved glomerular and tubular structures in mice (Figure 5H). Collectively, these findings indicate that POP exerts a mitigating effect on renal injury in T2DM mice.

### 3.8 Effect of POP on oxidative stress in T2DM mice

**Figure 6:**
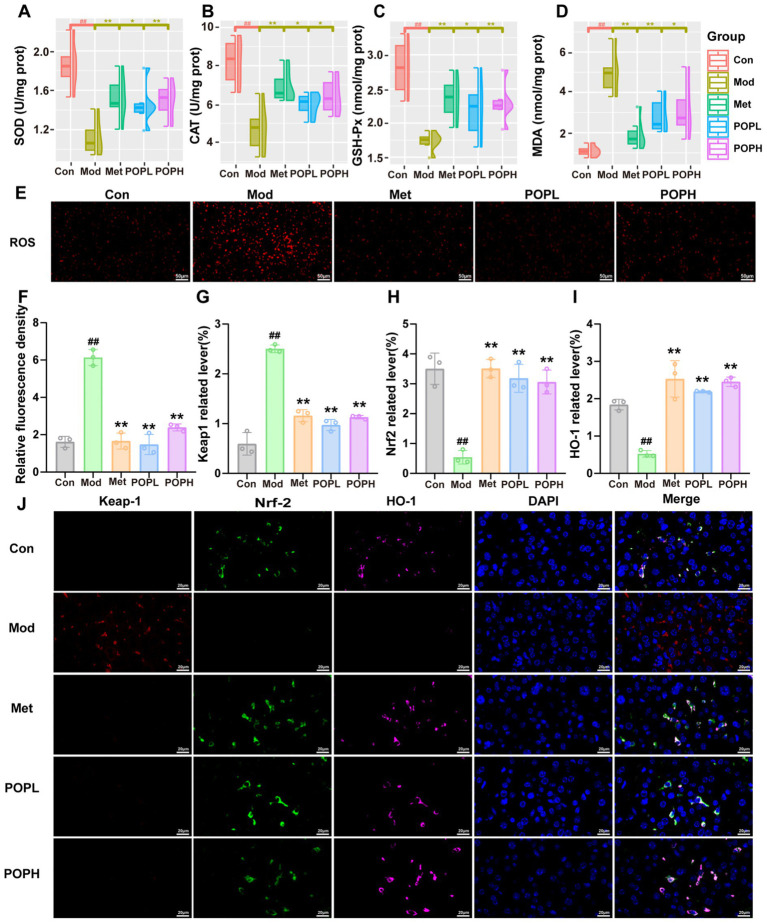
Effects of POP on oxidative stress and Nrf2-Keap1 pathway in T2DM mice. **(A)** SOD. **(B)** CAT. **(C)** GSH-Px. **(D)** MDA. **(E)** *In situ* DHE staining for ROS detection. **(F)** Relative fluorescence density of ROS. **(G)** Keap1 related lever. **(H)** Nrf2 related lever. **(I)** HO-1 related lever. **(J)** Immunofluorescence staining of Keap1 (red), Nrf2 (green) and HO-1 (purple) in liver. Data are expressed as mean ± SD. #*p* < 0.05, ##*p* < 0.01, compared with the Con group; *p < 0.05, **p < 0.01, compared with the Mod group.

In this study, we assessed the *in vivo* oxidative stress levels in each group of mice by measuring five oxidative stress markers: ROS, SOD, CAT, GSH-PX, and MDA in the liver of each group. The results (Figures 6A–F) indicated that the combined induction of HFD/STZ led to a significant increase (*p* < 0.01) in ROS levels in the livers of T2DM mice compared to the Con group, suggesting that the livers of T2DM mice experienced significant oxidative stress injury. Furthermore, compared to the Con group, the activities of SOD, CAT, and GSH-PX were significantly decreased (*p* < 0.01), while the MDA content was significantly increased (*p* < 0.01) in the livers of the Mod group mice. Notably, these changes were significantly reversed (*p* < 0.01) following POP intervention. These results suggest that POP exerts a significant antioxidative stress effect.

To further investigate the mechanism underlying the antioxidative stress capability of POP, this study measured the expression of Nrf2 and key proteins involved in the Nrf2 signaling pathway through immunofluorescence analysis. The results from the immunofluorescence technique (Figures 6G–J) indicated that, compared to the Con group, the red fluorescence intensity of Keap1 in the livers of mice from the Mod group was significantly enhanced (*p* < 0.01). In contrast, the green fluorescence intensity of Nrf2 and the pink fluorescence intensity of HO-1 were both significantly reduced (*p* < 0.01). Notably, POP treatment significantly increased the protein expression of Nrf2 in the livers of T2DM mice (*p* < 0.01) while significantly decreasing the protein expression of Keap1 (*p* < 0.01). These results suggest that POP may activate the Nrf2 signaling pathway, promote Nrf2 nuclear translocation, accelerate Keap1 degradation, and subsequently enhance the expression of downstream HO-1, thereby inhibiting oxidative stress induced liver injury in T2DM mice.

## 4 Discussion

### 4.1 Principal findings and integration with the hypothesis

This study presents the first comprehensive evidence that a hydrolysate derived from POP, predominantly composed of low-molecular-weight oligopeptides rich in leucine and valine, confers significant protective effects against HFD/STZ-induced T2DM in mice. Our initial hypothesis that POP would alleviate T2DM symptoms was substantiated, as evidenced by its remarkable capacity to improve hyperglycemia, insulin resistance, lipid metabolism disorders, and histopathological damage in key metabolic organs (pancreas, liver, and kidney). Crucially, we identified that the activation of the Nrf2/Keap1 antioxidant signaling pathway and the subsequent mitigation of systemic oxidative stress constitute a central mechanism underlying POP’s therapeutic efficacy.

### 4.2 POP composition and its implications for bioactivity

Our LC–MS/MS analysis indicates that POP is predominantly composed of oligopeptides (<1.5 kDa), which aligns with the characteristic profile of bioactive peptide hydrolysates recognized for their enhanced intestinal absorption and bioavailability compared to intact proteins or free amino acids (23, 24). The notable abundance of hydrophobic amino acids, particularly leucine and valine, is of particular significance. Leucine has been independently reported to stimulate insulin secretion and modulate mTOR signaling pathways, while valine and other branched-chain amino acids (BCAAs), although often linked to insulin resistance when present in excess, can exhibit insulin-sensitizing effects when incorporated into specific peptide sequences (25, 26). This observation suggests that the unique peptide profile of POP, rather than merely its overall composition, may be crucial for its observed bioactivity. The specific peptide sequences responsible for DPP-IV inhibition or Nrf2 activation remain to be elucidated, representing a critical area for future research aimed at identifying lead compounds (27, 28).

### 4.3 Amelioration of glucose homeostasis and insulin sensitivity: beyond direct effects

The significant improvements observed in FBG, HbA1c, OGTT, and ITT following POP treatment unequivocally demonstrate its potent antihyperglycemic and insulin-sensitizing effects (28–30). The increase in HOMA-*β* and the amelioration of islet morphology indicate a direct protective effect on pancreatic β-cells, potentially shielding them from STZ-induced cytotoxicity and metabolic stress (31). This finding is noteworthy, as the preservation of functional β-cell mass is a cornerstone of T2DM management. While our data suggest that the Nrf2-mediated reduction of oxidative stress is a key mechanism enhancing insulin signaling, we cannot dismiss the potential contributions from other pathways. For instance, certain dietary peptides are known to influence incretin secretion (GLP-1, GIP) or modulate the activity of key enzymes such as DPP-IV and sodium-glucose cotransporters (SGLTs) (5, 28). Investigating whether POP engages these additional mechanisms may reveal a multi-targeted mode of action.

### 4.4 Modulation of lipid metabolism and multi-organ protection

The alleviation of dyslipidemia, characterized by reduced levels of TC, TG, and LDL-C, alongside a marked improvement in liver function markers such as ALT, AST, and ALP underscore the beneficial effects of POP on systemic metabolism. The liver serves as the central hub for both glucose and lipid metabolism, making it a primary target in T2DM (32). The reduction in hepatic steatosis, evidenced by H&E and PAS staining, coupled with the enhanced glycogen storage capacity, suggests that POP improves hepatic insulin sensitivity. This likely disrupts the vicious cycle where in hepatic IR promotes gluconeogenesis and dyslipidemia, which in turn exacerbates IR (33). Although the improvement in renal function parameters and glomerular structure is modest, it indicates POP’s potential to slow the progression of diabetic nephropathy, a common and devastating complication. The protective effects observed across multiple organs suggest a systemic effect, likely mediated by the reduction of circulating oxidative stress and inflammatory mediators.

### 4.5 The central role of Nrf2/Keap1 pathway activation in mediating POP’s effects

The mechanistic insight from this study is the activation of the Nrf2/Keap1 pathway. The significant downregulation of Keap1, along with the nuclear translocation of Nrf2 and the upregulation of its downstream target HO-1, provides robust evidence that POP functions as a potent Nrf2 activator (34, 35). This finding is particularly significant given that chronic oxidative stress, induced by hyperglycemia, represents a fundamental pathophysiological characteristic of T2DM, contributing to both IR and *β*-cell dysfunction (36). By enhancing the endogenous antioxidant defense system evidenced by elevated levels of SOD, CAT, and GSH-Px—and diminishing oxidative damage, as indicated by reduced levels of MDA and ROS, POP fosters a less hostile metabolic environment. This likely facilitates improved insulin signal transduction in insulin-sensitive tissues and reduces apoptosis in β-cells (37). Our findings position POP alongside other natural Nrf2 activators under investigation for T2DM management, with the unique advantage of being a peptide-based extract derived from a traditional anti-diabetic medicinal food.

### 4.6 Clinical implications and translational considerations

The present findings suggest potential practical value for POP as a functional food ingredient or nutraceutical candidate for metabolic health. In a clinical context, such products may be most relevant for individuals with insulin resistance, impaired glucose tolerance, or early stage T2DM, where long-term dietary interventions are often recommended in parallel with standard care (38). Importantly, POP improved glycemic control, lipid profiles, and multi-organ histopathology in this model, indicating that its benefits may extend beyond glucose lowering to broader cardiometabolic risk-related endpoints (39).

Nevertheless, several translational steps are required before human application. These include establishing product standardization (e.g., peptide fingerprints and stability) (40), clarifying oral bioavailability and effective human-equivalent dosing, and evaluating safety through sub-chronic/chronic toxicology and, ultimately, well-designed early-phase clinical trials.

### 4.7 Limitations and future perspectives

Despite the promising findings, this study has several limitations. First, although the activation of the Nrf2 pathway is strongly correlated with the therapeutic effects, definitive proof of causality necessitates interventional experiments utilizing Nrf2 inhibitors or knockout animal models. Second, the specific peptide sequences within POP that are responsible for the observed effects remain unidentified. Future research should prioritize the isolation and characterization of the most active peptides, their synthesis, and the validation of their efficacy and mechanisms. Third, the potential influence of POP on gut microbiota, increasingly recognized as a key player in T2DM and a modulator of host metabolism, was not investigated, representing an exciting avenue for future exploration. Finally, the pharmacokinetics and long-term safety profile of POP must be established before any clinical applications can be considered.

## 5 Conclusion

In conclusion, this study reveals POP as a novel, multi-faceted agent in the preclinical treatment of experimental T2DM. Its efficacy is attributed to a combination of improved glycemic control, reduced insulin resistance, corrected dyslipidemia, and protection of vital organs from diabetic injury. The activation of the Nrf2/Keap1 signaling pathway and the subsequent reduction of oxidative stress emerge as a central mechanism of action. These findings not only provide a scientific basis for the traditional use of *Polygonatum odoratum* in diabetes but also emphasize its protein-derived components as a valuable resource for the development of innovative functional foods or nutraceuticals aimed at the prevention and adjunctive management of T2DM.

## Data Availability

The original contributions presented in the study are included in the article/Supplementary material, further inquiries can be directed to the corresponding authors.

## Glossary

ALT: Alanine aminotransferase
ALP: Alkaline phosphatase
AST: Aspartate aminotransferase
CAT: Catalase
FBG: Fasting blood glucose
GSH-PX: Glutathione peroxidase
HbA1C: Hemoglobin A1C
HDL-C: High-density lipoprotein cholesterol
HFD: High-fat diet
HOMA-*β*: Homeostasis model assessment of β-cell function
HOMA-IR: Homeostasis model assessment of insulin resistance
INS: Insulin
ITT: Insulin tolerance test
Keap1: Kelch-like ECH-associated protein 1
LC–MS/MS: Liquid chromatography–tandem mass spectrometry
LDL-C: Low-density lipoprotein cholesterol
MDA: Malondialdehyde
Nrf2: Nuclear factor erythroid 2-related factor 2
OGTT: Oral glucose tolerance test
POP: Polygonatum odoratum protein hydrolysate
ROS: Reactive oxygen species
SOD: Superoxide dismutase
STZ: Streptozotocin
TC: Total cholesterol
TG: Triglyceride
T2DM: Type 2 diabetes mellitus
